# Chylothorax vs. Pseudochylothorax: Cholesterol Crystal Finding in Pleural Effusion

**DOI:** 10.1155/crii/2358654

**Published:** 2026-10-20

**Authors:** Elena Pizarro Peña, Manuel Ruiz Artero, Amanda Jiménez García, Carmen García Rabaneda, Félix Gascón Luna

**Affiliations:** ^1^ Clinical Analysis Department Hospital Valle de los Pedroches Pozoblanco Córdoba Spain

**Keywords:** cholesterol, chylothorax, pseudochylothorax, rheumatoid arthritis

## Abstract

Chylothorax and pseudochylothorax are both rare forms of pleural effusion characterized by a milky appearance of the pleural fluid. Despite their similarities, they have distinct etiologies, clinical implications, and medical management strategies. Pleural fluid analysis of cholesterol and triglyceride levels is crucial to differentiate between them, establish a proper diagnosis, and guide clinical evaluation and treatment. This case report describes an uncommon presentation of pseudochylothorax associated with long‐standing rheumatoid arthritis (RA) in a patient with no prior history of pleural disease.

## 1. Case Report

An 82‐year‐old male, who worked as a quarry worker for 30 years and has a history of hypertension and rheumatoid arthritis (RA), was admitted to the emergency department presenting with dyspnea lasting approximately 3 months. No history of tuberculosis or other respiratory tract infections was reported. His usual treatment consisted of methotrexate (20 mg/week), oral iron, and folic acid supplementation for the last 5 years. Upon examination, he reported no chest pain, cough, or fever.

Chest ultrasound shows a dense fluid collection confirmed by computed tomography, with no sign of pleural thickening, masses or parenchymal lesions. Radiological evaluation demonstrated a large, homogenous pleural effusion occupying the dependent portions of the hemithorax, with a high attenuation content on computed tomography consistent with proteinaceous or lipid‐rich fluid. No pleural nodularity, thickening, or enhancement was identified, and there were no mediastinal masses, lymphadenopathy, or parenchymal lesions to suggest malignancy or lymphatic obstruction.

Informed consent was obtained to drain the pleural effusion via thoracentesis for further laboratory analysis. The results of the complete biochemical and cytological analyses are shown in Table 1.

**Table 1:** Results of pleural effusion analysis.

Test	Results	Reference values
Appearance	Turbid	Light yellow, clear
pH	7.170	7.60–7.65
Glucose	4	60–100 mg/dL (similar to plasma)
Total protein	5.0	1–2 g/dL
Lactate dehydrogenase (LDH)	1753	300–500 (<65% superior range of plasma levels)
Erythrocytes	30	0
Leukocytes	0	0
Other malignant cells	0	0
Adenosine deaminase	17.5	4–35 U/L

As shown, fluid analysis revealed the absence of leukocytes and other cells of interest. Cultures and Gram stains were negative. Acid‐fast bacilli (AFB) staining for mycobacteria was also negative, and the adenosine deaminase result was normal; consequently, tuberculous infection was initially excluded.

Unfortunately, no concurrent serum sample was submitted to the laboratory to definitively establish the classification as an exudate or transudate using light’s criteria. However, the pleural fluid profile was suggestive of an exudative effusion despite the absence of paired serum values. Findings that strengthen the pseudochylothorax diagnosis included the presence of cholesterol crystals under polarized light, low triglyceride levels, absence of chylomicrons, and an inflammatory exudative biochemical profile with markedly elevated LDH and very low glucose. Although lipoprotein electrophoresis is the gold standard, chylomicron absence was determined by Sudan III fat stain and further microscopy visualization due to the availability and quick results of the technique. Taken together with the clinical context of long‐standing RA and lack of strong evidence of infectious and malignant causes, these findings provide enough diagnostic confidence for pseudochylothorax despite incomplete biochemical pairing with serum samples.

Biochemical analysis shows enough evidence to classify the effusion as an exudate due to the combination of pH, total protein, LDH, and glucose results. As it is stated before in this paper, the most relevant finding corresponds to a high amount of cholesterol crystals with their characteristic flat and rhomboid form and sharp edges. Cholesterol crystals can be observed under polarized light or through microscopy using an automated analyzer (Figure 1).

**Figure 1:**
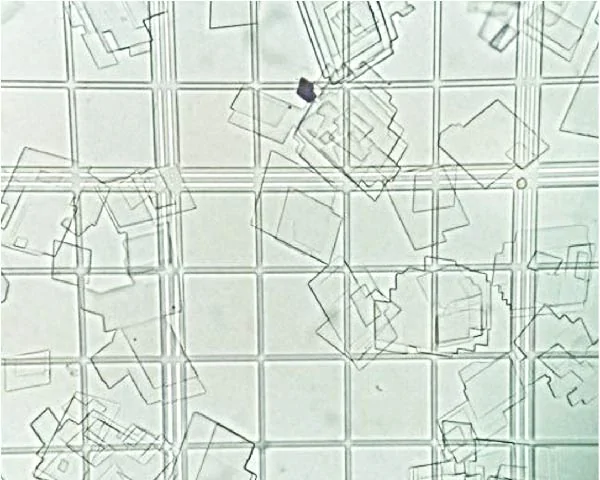
Cholesterol crystals in pleural fluid.

Given the initial differential diagnosis of chylothorax, additional cholesterol and triglyceride measurements were performed on the pleural fluid, yielding values of 125 mg/dL and 19 mg/dL, respectively. These results excluded chylothorax, and considering TB unlikely (although not fully excluded) based on the overall clinical, microbiological, and biochemical findings, this pleural fluid presentation would be compatible with pseudochylothorax associated with RA, a significantly less common clinical condition.

## 2. Discussion

Pseudochylothorax is a type of pleural effusion associated with long‐standing tuberculosis and autoimmune diseases such as RA. It is characterized by the presence of cholesterol crystals, which are pathognomonic, although cholesterol levels are usually higher (>200 mg/dL) than in the presented case. However, this finding is not unusual, as reference thresholds vary across publications, and cases of pseudochylothorax with cholesterol levels lower than 200 mg/dL are documented [1, 2].

The most frequent causes of pseudochylothorax include tuberculosis, RA, and parasitic infections (such as paragonimiasis or echinococcosis), as well as occasional cases secondary to surgical trauma or neoplasia. Although the exact pathophysiology is not fully elucidated, pseudochylothorax associated with RA is assumed to be produced by impaired lipid absorption caused by chronic pleural thickening, resulting in the accumulation of lipids within the pleural cavity. The prevalence of this entity is quite low; some authors report that fewer than 5% of patients with long‐standing RA develop a pseudochylothorax [3, 4].

In pseudochylothorax, imaging findings are typically non‐specific but may show features of chronicity, including long‐standing effusions, loculation, and occasionally pleural thickening or calcification in advanced disease. In this case, the absence of structural pleural abnormalities supported a non‐neoplastic inflammatory etiology, in keeping with rheumatoid pleuritis‐associated pseudochylothorax.

On the contrary, chylothorax is characterized by the presence of chylomicrons after centrifugation and high levels of triglycerides (>110 mg/dL). In addition, the pleural fluid‐to‐serum triglyceride ratio should be >1 and the pleural fluid‐to‐serum cholesterol ratio less than 1 [5]. As noted, no concurrent serum sample was sent to the laboratory to evaluate these ratios. However, the fluid analysis results—specifically, the presence of cholesterol crystals alongside low glucose and low triglyceride levels—effectively excluded chylothorax [2, 4].

The most common etiology of chylothorax involves direct injury to the thoracic duct resulting from cardiac or thoracic surgery. Postoperative complications (particularly after esophageal interventions) and nontraumatic causes such as malignancies (especially lymphoma), congenital lymphatic abnormalities, or parasitic infections can cause disruption or obstruction of the thoracic duct, resulting in the leakage of chyle into the pleural cavity [6].

The diagnosis in both cases is established via pleural effusion analysis. The presence of cholesterol crystals under polarized light has a 90% sensitivity for pseudochylothorax [7]. Cholesterol and triglyceride quantification, alongside the evaluation of chylomicrons after centrifugation, allows clinicians to reach a definitive diagnosis. The macroscopic appearance of the fluid also provides valuable diagnostic clues. The expected lipid values and composition of both fluids are summarized in Table 2.

**Table 2:** Main characteristics of chylothorax and pseudochylothorax fluid.

Characteristics	Chylothorax	Pseudochylothorax
Cause	Trauma, lymphoma, surgical intervention	Chronic diseases (tuberculosis, rheumatoid arthritis)
Composition	High triglyceride levels and chylomicrons	High cholesterol levels, low triglyceride levels
Appearance	Milky	Turbid or milky. No chylomicrons after centrifugation
Triglyceride	>110 mg/dL	<50 mg/dL
Cholesterol	<50 mg/dL	>200 mg/dL

Therapeutic management of pseudochylothorax consists of therapeutic thoracentesis and targeted treatment of the underlying disease. Surgical intervention is reserved only for recurrent and symptomatic cases [8]. An indwelling pleural catheter or surgical decortication can be utilized to significantly reduce the symptoms. In RA‐associated cases, corticosteroids and/or disease‐modifying antirheumatic drugs (DMARDs) like methotrexate should be optimized. Treatment follow‐up with serial imaging and clinical symptom monitoring is essential to ensure disease remission. Biologic therapies, such as adalimumab or infliximab, have also proven effective in managing long‐standing RA complications. The patient in this case was successfully treated with an increased dose of methotrexate (30 mg/week), close clinical monitoring over 3 months, and the addition of adalimumab (40 mg every 2 weeks), resulting in complete remission of respiratory symptoms.

In the event of chylothorax, conservative management is the primary approach. Thoracentesis is indicated in symptomatic patients to relieve chest pain and dyspnea. Dietary fat restrictions (e.g., a low‐fat diet supplemented with medium‐chain triglycerides [MCTs]) are established to maintain low triglyceride levels in the pleural fluid [9], and total parenteral nutrition (TPN) may be required in severe cases. Underlying conditions (such as lymphoma or traumatic injuries) must be addressed to resolve the effusion definitively and prevent recurrence. If chylothorax is caused by a luminal perforation (e.g., esophageal rupture), urgent surgical repair is mandatory, alongside broad‐spectrum antibiotic coverage to prevent secondary mediastinitis or empyema [4, 10]. Therapeutic management is resumed in Tables 3 and 4.

**Table 3:** Therapeutic management of pseudochylothorax.

Chronic pleural effusion → fluid analysis
↓
High cholesterol + low TG → pseudochylothorax
↓
Identify cause (TB/RA)
↓
Treat underlying disease
↓
Symptomatic?
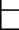 No → observe Yes → thoracocentesis
↓
Recurrent?
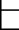 Yes → pleural catheter/decortication No → follow‐up

**Table 4:** Therapeutic management of chylothorax.

Milky fluid → check triglycerides
↓
>110 mg/dL → chylothorax
↓
Identify cause
↓
Initial management:
‐ Drainage
‐ Dietary modification (low‐fat, MCT diet)
‐ TPN if severe
↓
Persistent leak?
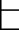 No → continue conservative Yes →
‐ Octreotide
‐ Thoracic duct ligation
‐ Interventional embolization

## 3. Conclusions

Chylothorax and pseudochylothorax are distinct causes of lipid‐rich pleural effusions that can appear macroscopically similar but differ fundamentally in their etiology, pathophysiology, and management. Chylothorax results from the disruption or obstruction of the thoracic duct, leading to the leakage of chyle into the pleural space. This occurs secondary to trauma (particularly iatrogenic injury following thoracic surgery), malignancy (most commonly lymphoma), or less frequently, congenital abnormalities and infections. In contrast, pseudochylothorax is a consequence of chronic pleural inflammation, classically associated with long‐standing tuberculosis and RA, where chronic fluid stasis leads to cellular degeneration and the accumulation of cholesterol.

Biochemical analysis remains the cornerstone of differentiation: chylothorax is characterized by elevated triglyceride levels (>110 mg/dL) and the presence of chylomicrons, whereas pseudochylothorax demonstrates elevated cholesterol levels (>200 mg/dL), low or normal triglycerides, and the pathognomonic presence of cholesterol crystals. While both entities typically satisfy exudative criteria, their clinical contexts and imaging findings differ; pseudochylothorax is frequently accompanied by structural features of chronicity, such as pleural thickening, calcification, and loculation. This distinction is clinically vital: chylothorax often requires prompt intervention (including dietary modification, specialized drainage, or surgical ligation), whereas pseudochylothorax is primarily managed conservatively by targeting the underlying chronic inflammatory disease and reserving drainage for symptomatic relief.

## Funding

No funding was received for this study.

## Ethics Statement

Written and verbal informed consent was obtained from the patient for the diagnostic and therapeutic procedures, as well as for the publication of this case report and its accompanying clinical data. According to local institutional policies, ethical committee approval is not required for single‐patient case reports involving standard diagnostic and therapeutic procedures, provided that informed consent is obtained and patient anonymity is strictly preserved. Laboratory samples were analyzed, stored, and discarded in accordance with the hospital ethical and environmental safety policies.

## Conflicts of Interest

The authors declare no conflicts of interest.

## Data Availability

The data that support the findings of this study are available from the corresponding author upon reasonable request.

## References

[1] Lama A. , Ferreiro L. , and Toubes M. E. , et al.Characteristics of Patients With Pseudochylothorax: A Systematic Review, Journal of Thoracic Disease. (2016) 8, no. 8, 2093–2101, 10.21037/jtd.2016.07.84. PMC4999702 27621864

[2] Hooper C. , Lee Y. C. G. , Maskell N. , and on behalf of the BTS Pleural Guideline Group , Investigation of a Unilateral Pleural Effusion in Adults: British Thoracic Society Pleural Disease Guideline 2010, Thorax. (2010) 65, no. Suppl 2, ii4–ii17, 10.1136/thx.2010.136978. 20696692

[3] Yokosuka T. , Suda A. , and Sugisaki M. , et al.Rheumatoid Pleural Effusion Presenting as Pseudochylothorax in a Patient Without Previous Diagnosis of Rheumatoid Arthritis, Respiratory Medicine Case Reports. (2013) 10, 37–9, 10.1016/j.rmcr.2013.09.003. PMC3920448 26029510

[4] Rosa R. , Maia D. , Caires N. , Gerardo R. , Gonçalves I. , and Cardoso J. , Pseudochylothorax Combined With Spontaneous Pneumothorax: Case Report of a Rare Complication of Rheumatoid Arthritis, Case Reports in Medicine. (2018) 2018, 10.1155/2018/7846962, 7846962. PMC5937586 29849661

[5] Braun C. M. and Ryu J. H. , Chylothorax and Pseudochylothorax, Clinics in Chest Medicine. (2021) 42, no. 4, 667–675, 10.1016/j.ccm.2021.08.003. 34774173

[6] Riley L. E. and Ataya A. , Clinical Approach and Review of Causes of a Chylothorax, Respiratory Medicine. (2019) 157, 7–13, 10.1016/j.rmed.2019.08.014. 31454675

[7] Porcel J. M. , Pardina M. , and Gatius S. , Rheumatoid Pseudochylothorax, Archivos de Bronconeumologia. (2020) 56, no. 10, 666–667, 10.1016/j.arbr.2020.08.002. 31669159

[8] Singh S. K. , Mathur K. , and Agarwal A. , et al.A Rare Case of Boerhaave Syndrome With Chylothorax Managed by an Endoscopic Over-the-scope Clip, ACG Case Reports Journal. (2025) 12, no. 11, 10.14309/crj.0000000000001869. PMC12574519 41181618

[9] Nyatsambo C. , Moeng M. S. , and Ngwisanyi W. , A Case Report on Chylothorax Post-Penetrating Chest Trauma, International Journal of Surgery Case Reports. (2023) 110, 10.1016/j.ijscr.2023.108637, 108637. PMC10428025 37572469

[10] Bhatnagar M. , Fisher A. , Ramsaroop S. , Carter A. , and Pippard B. , Chylothorax: Pathophysiology, Diagnosis, and Management-A Comprehensive Review, Journal of Thoracic Disease. (2024) 16, no. 2, 1645–1661, 10.21037/jtd-23-1636. PMC10944732 38505027

